# Azoturia, More Especially with Reference to Its Nomenclature and Pathology

**Published:** 1886-01

**Authors:** Richard W. Burke

**Affiliations:** Army Veterinary Department, Cawnpore, India


### ART. IV. — AZOTURIA, MORE ESPECIALLY WITH REFERENCE TO ITS NOMENCLATURE AND PATHOLOGY.

BY RICHARD W. BURKE, M.R.C.V.S.
*Army Veterinary Department, Cawnpore, India.*

SYNONYMS. — Hæmoglobinuria (Bollinger), Hysteria (Haycock), Azotœmia, Acute Uræmia (Walley), Neurasthenia Acuta, or Acute Nerve-Tire (Burke).

Various theories have been advanced to explain the pathology of this affection, and opinions still appear divided among the various observers. The most favorite are, renal inadequacy and liver derangement; but the explanations given by supporters of either theory appear to be a little conflicting. Their contention, if we understand their position aright, is that in this disease there is an imperfect conversion of albuminous substances, due to functional disorder of the liver, or to excessive supply of albumen, or to both these factors, which leads to an excess of urates in the blood, defective elimination by the kidneys, and an increase in the quantity of uric acid and urates in the blood. The admission that pre-existing defective capacity of the kidneys or disease of the liver will promote the development and accelerate the symptoms of Azoturia becomes an unavoidable corollary.

But the almost complete exclusion of what follows in the course of the above changes, in the production and distribution of the morbid phenomena, introduces difficulties which to me appear to have been hitherto left unexplained, and which may be called the alpha and omega of this whole process. The central fact remains, therefore, very nearly where it always was, since the main points unfolded by the above theories, and examined and re-examined by successive writers, have made little progress in comprehending what solutions of the problem are justifiable and true. Consequently we receive from writers an elaboration of their own hobbies, or whatever it may please each inquirer to call the result of his own investigation. Such theories, even though they may be universally accepted as a working hypothesis, yet show there is much need for an objection to them.

There are ample reasons for thinking that changes in the nervous centres determine the locality of each symptom in Azoturia, while changes in the relation of the blood, liver, and kidneys determine its effects. We may adopt for ourselves, then, the *neurotic theory*, since disturbances in the nervous system, in some form and part, may be regarded as a factor in every case of Azoturia. Dr. Laycock's "Lectures on Diseases of Organs and Tissues as influenced by the nervous system" bear directly upon this subject of Azoturia, and I would recommend their perusal in this connection.

We know how functional derangement produces certain definite conditions of temperament, or rather we are sure as of anything that "certain constitutions are prone to functional derangements of certain glandular organs, both secretive and eliminative, which alter the actual constituent normal condition of the blood," which is made manifest either in an objective or subjective manner ; and there can be no doubt that cause and effect, and effect and cause, both work together in the production of that unstable and irritable state of the nerve centres, which tends to a pathological state known as "Azoturia" in the horse. From an analysis of all the symptoms noticed in this disease I am confident we can find an explanation for the majority, if not all of them, which is especially marked out as nervous or neurotic.

With reference to the term Acute Uræmia, which implies that the blood is the starting point of the disease, it is known that numerous experiments performed on dogs and rabbits have proved that the subcutaneous injection of varying quantities of urea is followed by no one symptom, or symptoms, belonging to the disease which has been named by veterinarians *Azoturia;* while the experiments of Grèhaut and Quinquad have proved that similar injections of urea, equal to one-hundredth of the weight of the body, are always followed by death, without producing any symptoms of uræmia in the above animals. The presence of a large quantity of urea in the blood of animals does not seem to exert any injurious influence in producing the symptoms of this disease.

The presence of urates in the urine is secondary, — a result of organic changes due to altered innervation ; whilst albumin in the urine is not always present, and may be frequently produced, not only in the urine, but in the saliva and some other secretions and excretions, by the injection of various therapeutic agents. Semmola, * Vulpian, Dessales and others have shown that after hypodermic injections of pilocarpine, for example, the saliva, bile, and other secretions become richer in albuminoid substances coagulable by nitric acid, though none was to be found before the injections. In nephritis, in paralysis agitans, and other nervous disorders, albumin has been traced more or less in all the secretions. M. Strauss rendered the urine of animal albuminous by pricking the fourth ventricle, or by injecting glucose under the skin, but the kidneys did not present any organic lesions.

The connection of nerve disorders with uterine complaints is well known, and this may again explain perhaps the relative frequency of Azoturia in mares as compared with geldings or stallions. It would be a great mistake, however, to conclude that there is any necessary or constant connection between the two ; for, although very frequently the nerve disorder has originated in connection with uterine disturbance, in a large proportion of the cases we have seen it has developed independent of it, as in males.

I have no hesitation in asserting it to be my firm belief that many of the incurable cases of progressive muscular atrophy (especially of the crural muscles and occasionally of the muscles of the scapular region), paralysis, and many other diseases of the nervous system, commence as Azoturia ; and

* Annali Univ. di Medicina, Feb., 1885.

when in this state are quite amenable to treatment. Death is usually the result of some sequela in severe cases.

What are the usual characteristics of such subjects? Can they be described as weak, debilitated, and weedy looking? Certainly not; and the experience of practitioners of human medicine agrees with our own in an especial manner; for Dr. J. Stretch Dowse* describes such patients as being usually "robust, stout, plethoric, and apparently cheerful." Dr. W. S. Playfair † writes that occasionally such patients are "overburdened with an excess of adispose tissue." At a recent meeting of the balneological section of the Gesellschaft für Heilkunde, held in Berlin this year, Dr. Joseph, of Landeck, read a paper upon this subject, describing the condition of neurasthenic patients as "robust, muscular, and ruddy complexioned."

Among the drugs which have given most relief in cases of Azoturia, and which have been found reliable in our hands, we find the following, and I enumerate them in the order of their value, namely: chloral, opium and morphia,‡ belladonna (Walley) and atropine, chloroform, bromide and iodide of potassium, ergot, turpentine, quinine, ether, alcohol and ammonia; and, I would add, local friction (*massage*) and electricity. The reflex influence of mechanical stimuli of the cutaneous nerves on the cerebro-spinal circulation is of considerable importance.

I am tempted to put forward the above facts, because it is one of the most remarkable instances of the strange and multiform phenomena which neurotic disease may present, which it has ever been the lot of veterinarians to witness. Coincident with nerve disorder is the change in the constitution of the blood, due to changes in the secretory and excretory organs, particularly the liver and kidneys. On the soil so prepared are often developed the graver symptoms, such as paresis, or paralysis, hyperæsthesia, disorder of motion, hystero-epileptic seizures, convulsions, and many others which must be familiar to us all in dealing with such cases. The muscular tremors,

* Dr. J. S. Dowse, "On Nerve-Prostration." 1880, p. 11.

† Dr. W. S. Playfair, "On Nerve-Prostration and Hysteria." 18[illegible]3, p. 91.

‡ Journal of Comparative Medicine and Surgery. April, 1885.

the cramp, the unsteady gait, the loss of balance, &c., so marked in many cases, may be due to the nerve current from the cerebellum in its descent to the muscles finding an obstacle in the spinal cord altered by the morbid process.

